# Leveraging cell-type-specific regulatory networks to interpret genetic variants in abdominal aortic aneurysm

**DOI:** 10.1073/pnas.2115601119

**Published:** 2021-12-20

**Authors:** Shining Ma, Xi Chen, Xiang Zhu, Philip S. Tsao, Wing Hung Wong

**Affiliations:** ^a^Department of Statistics, Stanford University, Stanford, CA 94305;; ^b^Department of Statistics, Pennsylvania State University, University Park, PA 16802;; ^c^Huck Institutes of the Life Sciences, Pennsylvania State University, University Park, PA 16802;; ^d^VA Palo Alto Epidemiology Research and Information Center for Genomics, VA Palo Alto Health Care System, Palo Alto, CA 94304;; ^e^Department of Medicine, Stanford University School of Medicine, Stanford, CA 94305;; ^f^Stanford Cardiovascular Institute, Stanford University School of Medicine, Stanford, CA 94305;; ^g^Bio-X Program, Center for Personal Dynamic Regulomes, Department of Biomedical Data Science, Stanford University, Stanford, CA 94305

**Keywords:** HiChIP, abdominal aortic aneurysm, cell-type-specific regulation, whole-genome sequencing, smooth muscle cells

## Abstract

Abdominal aortic aneurysm (AAA) is a common degenerative cardiovascular disease whose pathobiology is not clearly understood. The cellular heterogeneity and cell-type-specific gene regulation of vascular cells in human AAA have not been well-characterized. Here, we performed analysis of whole-genome sequencing data in AAA patients versus controls with the aim of detecting disease-associated variants that may affect gene regulation in human aortic smooth muscle cells (AoSMC) and human aortic endothelial cells (HAEC), two cell types of high relevance to AAA disease. To support this analysis, we generated H3K27ac HiChIP data for these cell types and inferred cell-type-specific gene regulatory networks. We observed that AAA-associated variants were most enriched in regulatory regions in AoSMC, compared with HAEC and CD4^+^ cells. The cell-type-specific regulation defined by this HiChIP data supported the importance of *ERG* and the *KLF* family of transcription factors in AAA disease. The analysis of regulatory elements that contain noncoding variants and also are differentially open between AAA patients and controls revealed the significance of the interleukin-6-mediated signaling pathway. This finding was further validated by including information from the deleteriousness effect of nonsynonymous single-nucleotide variants in AAA patients and additional control data from the Medical Genome Reference Bank dataset. These results shed important insights into AAA pathogenesis and provide a model for cell-type-specific analysis of disease-associated variants.

## Significance

Abdominal aortic aneurysm (AAA) is a common and severe disease with major genetic risk factors. In this study we generated enhancer-promoter contact data to identify regulatory elements in AAA-relevant cell types and identified changes in their predicted chromatin accessibility between AAA patients and controls. We integrated this information with disease-associated variants in regulatory elements and gene bodies to further understand the etiology and pathogenetic mechanisms of AAA. Our study combined whole-genome sequencing data with gene regulatory relations in disease-relevant cell types to reveal the important roles of the interleukin 6 pathway and *ERG* and *KLF* regulation in AAA pathogenesis.

Abdominal aortic aneurysm (AAA) is a prevalent cardiovascular disease and the tenth leading cause of death in Western countries (1). It is defined as an irreversible dilation of the infrarenal aorta to a diameter of 30 mm or more, increasing more than 50% compared with its normal caliber (2). AAA is a complex and severe disease affected by a combination of genetic (3), environmental (4), and lifestyle factors (5). The genetic factor in AAA is critical and the heritability has been estimated to be over 70% (6, 7). Therefore, understanding the genetic pathology of AAA will guide clinical decisions for therapeutic intervention and provide mechanistic insights into the genetic architecture and individual susceptibility to AAA.

Although several associations between AAA and single-nucleotide polymorphisms (SNPs) have been reported, standard genome-wide association studies (GWAS) face challenges to identify reliable and replicable risk loci due to the evident mutational heterogeneity in AAA (8). Most recently, a genome-wide association study in the Million Veteran Program (MVP) tested over 18 million variants in Veterans of European ancestry and identified 14 novel risk loci, bringing the total number of known significant AAA loci to 24 (9). However, current knowledge of its underlying genetics only explains a small fraction of AAA heritability and remains insufficient to guide early detection and clinical management.

Integration of cell-type-specific regulatory information with array-based GWAS data has shown that disease-associated variants often pinpoint perturbed regulatory modules that are highly specific to disease-relevant cell types or tissues (10). In contrast to genotyping arrays which rely on linkage disequilibrium (LD) to provide coverage of the entire genome, WGS data can efficiently capture rare variants and variants not in LD with genotyped SNPs. Therefore, combining whole-genome sequencing data with gene regulation data specific in AAA-relevant cell types is a promising direction to improve the understanding of AAA pathogenesis.

**Fig. 1.**
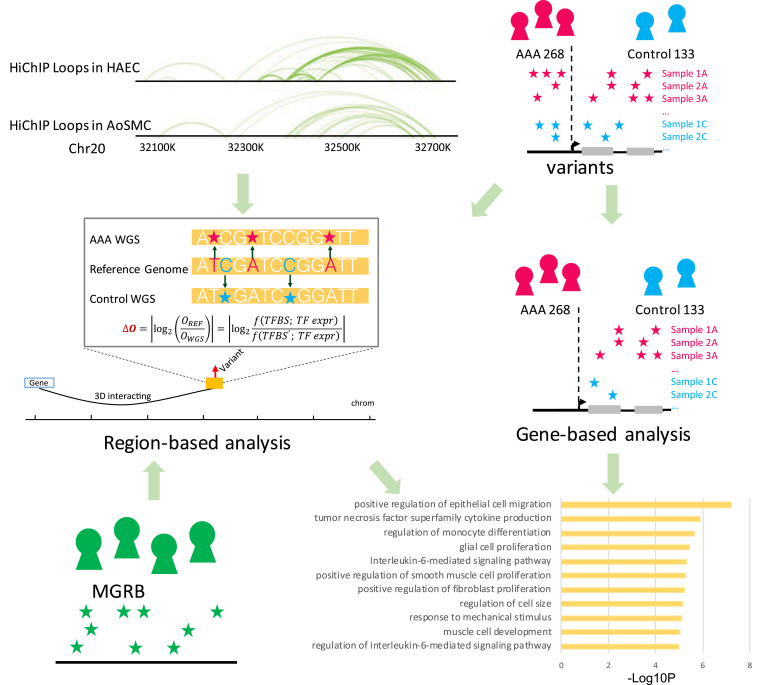
Overall study design of AAA. First, we defined cell-type-specific REs from the HiChIP experiments. We initially focused on the noncoding variants located in REs and performed region-based analysis, from which the REs with differential openness between AAA patients and controls were detected and the target genes of these REs were obtained through the HiChIP loops. Next, we quantified the gene mutation burden based on rare SNVs in gene coding regions. Additionally, we added MGRB data into the AAA analysis to increase statistical power. Through the above procedure, we successfully identified that the IL-6 pathway is related to AAA with strong statistical significance.

In order to infer gene regulatory networks in cell types relevant to AAA, we performed H3K27ac HiChIP experiments in aortic smooth muscle cells (AoSMC) and human aortic endothelial cells (HAEC) and combined this new data with existing gene expression and chromatin accessibility data. As a comparison, we also inferred CD4 T cell-specific gene regulatory network using existing data. We subsequently applied the constructed networks to AAA GWAS and gene expression data to derive cell-type-specific inference. Dataset S1 provides a summary of the datasets used in this study. Our analysis revealed high enrichment of AAA-associated variants in the regulatory elements (REs) of the AoSMC network and the role of *ERG* and the *KLF* family of transcription factors (TFs) in the differential expression of genes expressed in AAA. For the noncoding variant analysis, we performed regional analysis to predict the openness of REs based on the noncoding variants, from which the REs with differential openness between AAA patients and controls were detected and the target genes of these REs were obtained through the HiChIP loops. The evidence from noncoding variants is then combined with information on gene mutation burden based on rare single-nucleotide variants (SNVs) on coding regions (Fig. 1). A main finding of this analysis implicates the interleukin (IL)-6 pathway as relevant to AAA with strong statistical significance.

## Results

### Cell-Type-Specific Regulatory REs in HAEC and AoSMC.

To characterize the HiChIP-defined REs, we calculated the relationship between the chromatin accessibility of REs and the expression levels of their target genes. Based on the ATAC-seq (Assay for Transposase Accessible Chromatin with high-throughput sequencing) data from HAEC and AoSMC, we identified the cell-type-specific REs in HAEC and AoSMC (see *Materials and Methods*) and classified them into two categories: 1) open REs which overlapped with at least one ATAC-seq peak (OpenLoop) and 2) nonopen REs which did not overlap with any ATAC-seq peaks (NonOpenLoop). Next, we evaluated the expression of genes that had promoters linked to open REs, nonopen Res, or not linked to REs (NoLoop) (Table 1). We observed that the expression of genes whose promoters were linked to open REs tended to be higher than those linked with nonopen REs or not linked with REs. All *P* values of Wilcoxon rank-sum one-sided test were less than 2e-16 in both HAEC and AoSMC. We also noticed that the proportion of nonexpressed genes in the NonOpenLoop category was much higher in AoSMC than in HAEC (76% vs. 54%) (Dataset S2). To investigate this further, we collected 16,512 putative silencers in human blood vessel from SilencerDB (11). We detected the proportion of genes whose REs overlapped with silencers (directly) or linked with silencers by HiChIP loops (indirectly) (Dataset S2). Consistent with the fact that silencers are active REs and many of them reside in the open chromatin regions (12), we observed that the percentage of genes regulated by silencers was higher in the OpenLoop category compared to the NonOpenLoop category in both cell types. Moreover, the proportion of genes in the NonOpenLoop category regulated by silencers was much higher in AoSMC than in HAEC (34% vs. 23%) with Fisher’s exact test *P* value = 6e-12, which may explain in part the higher proportion of nonexpressed genes in AoSMC.

**Table 1.** Quantile expression of genes whose promoters are linked with open REs, nonopen Res, or not linked with HiChIP REs

			Quantile expression (FPKM)						
Cell type	Openness	No. of genes	5%	10%	25%	50%	75%	90%	95%
AoSMC	OpenLoop	8,693	0	0	0.17	5.36	17.61	47.03	90.74
	NonOpenLoop	1,280	0	0	0	0	0	10.95	26.05
	NoLoop	16,040	0	0	0	0.01	2.75	14.43	28.37
HAEC	OpenLoop	7,577	0	0	0.24	7.47	22.44	54.08	96.98
	NonOpenLoop	1,679	0	0	0	0	9.54	29.87	57.91
	NoLoop	16,617	0	0	0	0	3.09	17.02	30.28

### AAA-Associated Variants Are the Most Enriched in Regulatory Regions in AoSMC.

To test whether the REs defined from HiChIP data were enriched for genetic associations with AAA, we performed RSS-NET network enrichment analysis (12) based on the summary-level association data from the MVP AAA discovery analysis which contained ∼18 million variants from 7,642 AAA patients and 172,172 controls (9). RSS-NET is a Bayesian framework that integrates GWAS summary statistics with cell-type-specific regulatory networks to infer network enrichment. RSS-NET summarizes the network enrichment strength as a Bayes factor (BF). A large BF value indicates strong enrichment of genetic associations in a given network. We assessed enrichment of genetic associations with AAA in five HiChIP-based RE-target gene (TG) networks and one near-gene control network (see *Materials and Methods*). All five HiChIP networks were more enriched for genetic associations with AAA than the near-gene control network (Table 2), of which AoSMC HiChIP was the most enriched network for genetic associations with AAA (i.e., the largest BF value). The top ranked genes defined by RSS-NET in each network are shown in Dataset S3. The observed enrichment patterns were consistent across three enrichment models (M11, M12, and M1), validating the importance of cell-type-specific networks in genetic analysis of AAA.

**Table 2.** RSS-NET network enrichment

Networks	BF(M11:M0)	BF(M12:M0)	BF(M1:M0)
AoSMC_HiChIP_open	734.58	628.33	632.17
HAEC_HiChIP_open	214.81	188.39	187.84
AoSMC_HiChIP	1,704.83	1420.74	1,443.30
HAEC_HiChIP	53.38	47.23	46.97
CD4^+^ T_HiChIP	4.63	4.46	4.34
Near-gene	1.87	1.78	1.76

### Implication of the *KLF* and *ERG* Regulators in the Down-Regulation of Genes in AAA.

We utilized data from Biros et al. (13), which included 1,178 differentially expressed genes between AAA patients and healthy controls, of which 941 were down-regulated and 237 were up-regulated in AAA compared with controls. From HiChIP H3K27ac loops in HAEC and AoSMC we derived REs regulating differentially expressed genes and then intersected these with ATAC-seq peaks to obtain open REs of differentially expressed genes. We performed motif enrichment based on these REs with Homer (v4.11) (14). Motifs of the *KLF* family are enriched in open REs of AAA down-regulated genes in HAEC (fold change [FC] = 1.7, *P* = 1e-20, ranking = fifth among all motifs) and AoSMC (FC = 1.4, *P* = 1e-16, ranking = 10th). Moreover, we derived the target genes of the *KLF* family in CD4^+^ primary T cells predicted by the PECA (15) model, which inferred gene regulatory interactions using paired expression and accessibility data across diverse cellular contexts. Based on the REs identified this way in CD4^+^ T cells, we found that target genes of the *KLF* family were enriched in up-regulated genes in AAA with a *P* value less than 2e-16. Our result is consistent with the previous findings that *KLF* family members are involved in AAA formation (16–18) and further suggest that their roles may be different in AoSMC and HAEC as compared to CD4^+^ T cells.

In HAEC, the *ERG* motif is enriched in open REs of down-regulated genes in AAA patients (FC = 2.5, *P* = 1e-96, ranking = second). A known risk locus associated with AAA, the SNP rs2836411, is located within an intron of gene *ERG* (3, 9). Expression of *ERG* and rs2836411 were shown to be associated in an independent expression quantitative trait loci dataset derived from mammary artery tissue (19). The *ERG* gene encodes a transcriptional regulator protein which is normally present in hematopoietic and endothelial cells. Moreover, *ERG* plays a role in VEGF (vascular endothelial growth factor)-mediated vascular development and endothelial cell activation (20). Therefore, *ERG* may influence the development of AAA as a key mediator of vascular angiogenesis and inflammation. Our analysis not only corroborates these prior results but also provides insight into the target genes of these important regulators in a cell-type-specific manner. Interestingly, *ERG* had been shown to interact physically with *KLF* factors to regulate the *VEGF* receptor in vascular development in *Xenopus* (21).

### Identification of the IL-6 Pathway by Comparison of REs with Altered Openness between AAA and Controls.

To analyze the effect of noncoding rare variants between AAA patients and controls we applied the method OpenCausal (22) to interpret noncoding variants from whole-genome sequencing data in AAA and controls. OpenCausal attempts to interpret noncoding variants by using personal genomic sequences and reference context-specific expression profiles, which may reflect the change of chromatin accessibility caused by a variant. First, we used the variants located in the REs as the input to OpenCausal. Here, the variants were from whole-genome sequencing data of 268 AAA patients and 133 healthy controls (23), and the REs consisted of 1) HiChIP H3K27ac REs of AoSMC, HAEC, and CD4^+^ naïve T cells and 2) promoters which were defined as 2 kbp upstream of TSS.

For a given RE, we checked if the alteration of its openness was enriched in AAA individuals using the following approach. For 268 AAA patients and 133 healthy controls, we applied PLINK 1.9 (24) to obtain the frequency for each allele. Using the major allele for each variant, we constructed the genome of a “baseline individual.” For each RE, we used OpenCausal to obtain an openness score for each individual AAA patient and control, as well as for the baseline individual. We counted the number of individuals whose openness score for the RE was different from that of the baseline individual (nonbaseline openness) and applied Fisher’s exact test to measure if the individuals with nonbaseline openness for this RE were significantly more enriched in AAA patients than in controls. For a given RE, we also compared the openness score between 1) AAA patients with nonbaseline openness and 2) healthy controls with nonbaseline openness. We performed a Wilcoxon rank-sum test to detect if the openness score follows the same distribution in these two groups. We then used Fisher’s method to combine these two *P* values and to measure the statistical significance for altered accessibility of this RE in AAA patients. We repeated the above procedure for each RE. We linked these REs with target genes based on the information on RE–promoter interaction provided by the HiChIP data. As the alteration of one gene does not necessarily require all of its REs to be differentially open, we collected the top five REs with highest differential open scores for those genes regulated by multiple REs and then combined the effect of these REs using Fisher’s method. Cell-type-specific analysis was performed separately in AoSMC, HAEC, and CD4^+^ T cells, resulting in 232, 230, and 505 genes, respectively, with false discovery rate (FDR) <0.01 (control of FDR is based on the Benjamini–Hochberg [BH] procedure).

We performed Gene Ontology (GO) enrichment on these target genes of differential open REs using the ToppGene Suite (25). In AoSMC, the negative regulation of IL-6-mediated signaling pathway, regulation of IL-6-mediated signaling pathway, and IL-6-mediated signaling pathway were ranked among the top pathways (Table 3). In the IL-6-mediated signaling pathway, five genes (*IL6ST*, *SOCS3*, *MIR99A*, *MIRLET7C*, and *MIR125B2*) were the targets of nonbaseline open REs (i.e., RE with nonbaseline openness). *SOCS3* was detected to be significantly up-regulated in AAA patients in bulk RNA-sequencing (RNA-seq) data (13) and also up-regulated in the smooth muscle cell cluster from single-cell RNA-seq in AAA patients compared with control samples (26). In the negative regulation of IL-6-mediated signaling pathway, three microRNAs (miRNAs) (*MIRLET7C*, *MIR99A*, and *MIR125B2*) were the targets of nonbaseline open REs. Araujo et al. identified differentially expressed miRNAs by PCR array (27) and observed that all of *MIRLET7C*, *MIR99A*, and *MIR125B2* were significantly down-regulated in AAA patients compared to controls (*P* = 2e-3, 5e-3, and 3e-2 for *MIRLET7C*, *MIR125B2*, and *MIR99A*, respectively). Wanhainen et al. analyzed the expression of the 172 most commonly expressed miRNAs in plasma by real-time PCR from 169 AAA patients and 48 age-matched controls (28) and found *MIR99A* was significantly down-regulated in AAA patients (FDR *q*-value = 8e-6). Aortic expression of *MIRLET7C* was significantly down-regulated (*P* < 0.05) in a murine model of AAA (29). These observations are consistent with our finding that these miRNAs are linked more often to nonbaseline open REs in AAA patients. The GO enrichment results on target genes of differential open REs in HAEC and CD4^+^ T cells are shown in Dataset S4.

**Table 3.** GO enrichment of the target genes of the REs with differential openness in AoSMC

GO ID	GO term	Fold enrichment	*P* value	*q*-value Bonferroni	*q*-value FDR BH
GO:0010634	Positive regulation of epithelial cell migration	6.08	2.47E-08	8.77E-05	8.77E-05
GO:0045766	Positive regulation of angiogenesis	4.83	2.65E-07	9.40E-04	4.70E-04
GO:0045655	Regulation of monocyte differentiation	26.91	5.45E-07	1.93E-03	6.44E-04
GO:0070104	Negative regulation of IL-6-mediated signaling pathway	43.06	1.80E-06	6.39E-03	1.14E-03
GO:0048146	Positive regulation of fibroblast proliferation	9.13	1.83E-06	6.50E-03	1.14E-03
GO:0045638	Negative regulation of myeloid cell differentiation	7.10	1.93E-06	6.83E-03	1.14E-03
GO:0070103	Regulation of IL-6-mediated signaling pathway	34.45	3.60E-06	1.28E-02	1.53E-03
GO:0034763	Negative regulation of transmembrane transport	5.35	3.97E-06	1.41E-02	1.53E-03
GO:0070102	IL-6-mediated signaling pathway	16.56	4.27E-06	1.51E-02	1.53E-03
GO:0038034	Signal transduction in absence of ligand	7.83	4.74E-06	1.68E-02	1.53E-03
GO:0097192	Extrinsic apoptotic signaling pathway in absence of ligand	7.83	4.74E-06	1.68E-02	1.53E-03
GO:0002762	Negative regulation of myeloid leukocyte differentiation	10.34	5.34E-06	1.89E-02	1.58E-03
GO:0007219	Notch signaling pathway	4.33	6.85E-06	2.43E-02	1.84E-03
GO:0030857	Negative regulation of epithelial cell differentiation	9.75	7.27E-06	2.58E-02	1.84E-03
GO:1903707	Negative regulation of hemopoiesis	4.76	9.76E-06	3.46E-02	2.31E-03
GO:0010595	Positive regulation of endothelial cell migration	5.43	1.25E-05	4.44E-02	2.70E-03
GO:0030224	Monocyte differentiation	12.67	1.37E-05	4.87E-02	2.70E-03
GO:1903131	Mononuclear cell differentiation	12.67	1.37E-05	4.87E-02	2.70E-03

In addition to IL-6 related pathways, we found that *IL1B* was linked more often to nonbaseline open REs in AAA patients compared with healthy controls in AoSMC (adjusted *P* = 2e-4). *IL1B* has previously been found to be up-regulated in AAA patients by RNA-seq (adjusted *P* = 2e-3) (13) and quantitative PCR (*P* < 0.05) (26). These findings contribute to understanding the regulatory mechanisms of *IL1B* in AAA pathogenesis.

### Significance of the IL-6 Pathway Is Retained after Inclusion of Coding Variants.

After analyzing the effect of noncoding variants on AAA disease, we quantified the gene mutation burden based on the rare SNVs in protein-coding regions. To predict the deleteriousness of each nonsynonymous SNV, we applied a strategy similar to that in Li et al. (23), which was based on the average performance of three algorithms, VEST3 (30, 31), MetaLR (32), and M-CAP (33). We averaged the prediction scores to assess the deleteriousness of each nonsynonymous SNV for 17,443 protein-coding genes. For each of the 268 AAA patients and 133 controls, we repeated the above procedure and then obtained the deleteriousness scores for genes with a 17,443 × 401 matrix (see *Materials and Methods*).

For each gene, we performed a one-tailed *t* test to measure if the deleteriousness scores for each gene were significantly higher in AAA patients than in nonaneurysmal controls. We counted the number of genes with *t* test *P* ≤ 0.05 and the number of protein-coding genes in a GO term and then calculated the *P* value based on the Poisson distribution. For each GO term, we obtained two *P* values, one from the noncoding variants and the other from coding variants. We combined these two *P* values with Fisher’s method and ranked the GO terms based on the significance. In AoSMC, the IL-6-mediated signaling pathway, regulation of IL-6-mediated signaling pathway, and negative regulation of IL-6-mediated signaling pathway were ranked among the top pathways (Table 4). We detected *STAT1*, *GFI1*, and *PTPN11* in the IL-6 pathway with *P* value less than 0.05 from the coding region analysis. Moreover, *PTPN11* was significantly down-regulated in AAA patients compared with healthy controls.

**Table 4.** The combined enrichment analysis of noncoding and coding variants in AoSMC

GO ID	GO term	Fold enrichment	*P* value	*q*-value FDR
GO:0010634	Positive regulation of epithelial cell migration	1.84	5.84E-08	1.52E-04
GO:0071706	Tumor necrosis factor superfamily cytokine production	1.78	1.29E-06	1.67E-03
GO:0045655	Regulation of monocyte differentiation	4.87	2.25E-06	1.95E-03
GO:0014009	Glial cell proliferation	3.10	3.62E-06	2.26E-03
GO:0070102	IL-6-mediated signaling pathway	4.06	4.87E-06	2.26E-03
GO:0048661	Positive regulation of smooth muscle cell proliferation	2.01	5.40E-06	2.26E-03
GO:0048146	Positive regulation of fibroblast proliferation	2.43	6.09E-06	2.26E-03
GO:0008361	Regulation of cell size	1.77	7.36E-06	2.35E-03
GO:0009612	Response to mechanical stimulus	1.85	8.18E-06	2.35E-03
GO:0042692	Muscle cell development	1.45	9.45E-06	2.35E-03
GO:0070103	Regulation of IL-6-mediated signaling pathway	5.41	9.96E-06	2.35E-03
GO:0034763	Negative regulation of transmembrane transport	1.63	1.21E-05	2.60E-03
GO:1901888	Regulation of cell junction assembly	1.70	1.30E-05	2.60E-03
GO:1903557	Positive regulation of tumor necrosis factor superfamily cytokine production	2.43	1.55E-05	2.87E-03
GO:0030857	Negative regulation of epithelial cell differentiation	2.23	1.81E-05	3.14E-03
GO:0045638	Negative regulation of myeloid cell differentiation	1.14	2.38E-05	3.51E-03
GO:0038034	Signal transduction in absence of ligand	1.83	2.46E-05	3.51E-03
GO:1902107	Positive regulation of leukocyte differentiation	1.51	2.53E-05	3.51E-03
GO:0070104	Negative regulation of IL-6-mediated signaling pathway	5.21	2.57E-05	3.51E-03

### The Use of a Larger Sample of Control Individuals Led to a Further Increase in the Significance of the IL-6 Pathway.

The statistical power was limited by the small sample size of our WGS data for healthy controls. To alleviate this problem, we added data from the Medical Genome Reference Bank (MGRB) into our analysis. The MGRB is a large-scale comprehensive whole-genome dataset of confirmed healthy elderly individuals (34). Our AAA subjects were of European ancestry (> 90%) and the MGRB cohorts were non-Finnish European (∼97%). Here, we randomly selected 500 samples (Dataset S5) from the first release of the MGRB healthy elderly cohort, which contained 2,572 samples. We maintained the sex ratio of these 500 samples with that of our 268 AAA patients. We removed variants which appeared only in the MGRB samples but not in the AAA dataset. We then performed OpenCausal analysis with the 268 AAA patients, substituting the 500 MGRB samples for our original 133 controls. Similar to the previous noncoding region analysis, for each RE we obtained an openness score for each AAA and control individual, as well as for the reference genome. We counted the number of individuals whose openness score for the RE was different from that of the reference genome and then performed Fisher’s exact test and Wilcoxon rank-sum test. We used Fisher’s method to combine these two *P* values and to measure the statistical significance for altered accessibility of an RE in AAA patients. We then linked these REs with target genes based on the AoSMC HiChIP data. Similarly, we collected five REs with the highest differential open scores for those genes regulated by multiple REs and then combined the effect of these REs using Fisher’s method, obtaining 4,238 genes with FDR <0.01 in AoSMC. We overlapped these 4,238 genes with 232 genes identified by the original AAA dataset (268 AAA patients and 133 controls) and obtained 213 shared genes. Again, we performed GO enrichment on the 213 genes (Table 5) and found that the negative regulation of IL-6-mediated signaling pathway and regulation of IL-6-mediated signaling pathway were ranked among the top pathways with FDR <0.001. These results demonstrated that the use of a larger number of control individuals from MGRB could further increase the statistical significance of the IL-6 pathway in AAA disease.

**Table 5.** GO enrichment on overlapped genes in AoSMC

GO ID	GO term	Fold enrichment	*P* value	*q*-value Bonferroni	*q*-value FDR
GO:0010634	Positive regulation of epithelial cell migration	6.04	4.42E-08	1.28E-04	1.28E-04
GO:0045655	Regulation of monocyte differentiation	29.34	2.43E-07	7.02E-04	3.51E-04
GO:0045638	Negative regulation of myeloid cell differentiation	7.74	5.64E-07	1.63E-03	4.44E-04
GO:0048146	Positive regulation of fibroblast proliferation	9.96	6.15E-07	1.78E-03	4.44E-04
GO:0070104	Negative regulation of IL-6-mediated signaling pathway	46.95	9.43E-07	2.72E-03	5.45E-04
GO:0070103	Regulation of IL-6-mediated signaling pathway	37.56	1.89E-06	5.45E-03	8.60E-04
GO:0002762	Negative regulation of myeloid leukocyte differentiation	11.27	2.08E-06	6.02E-03	8.60E-04
GO:1903707	Negative regulation of hemopoiesis	5.18	2.58E-06	7.47E-03	9.33E-04
GO:0002761	Regulation of myeloid leukocyte differentiation	5.73	4.70E-06	1.36E-02	1.51E-03
GO:1903131	Mononuclear cell differentiation	13.81	6.23E-06	1.80E-02	1.64E-03
GO:0030224	Monocyte differentiation	13.81	6.23E-06	1.80E-02	1.64E-03
GO:0070228	Regulation of lymphocyte apoptotic process	8.80	7.79E-06	2.25E-02	1.88E-03
GO:0048145	Regulation of fibroblast proliferation	6.20	1.17E-05	3.37E-02	2.50E-03
GO:1902106	Negative regulation of leukocyte differentiation	6.14	1.23E-05	3.57E-02	2.50E-03
GO:0002902	Regulation of B cell apoptotic process	20.87	1.32E-05	3.82E-02	2.50E-03
GO:0048144	Fibroblast proliferation	6.03	1.38E-05	4.00E-02	2.50E-03
GO:0045656	Negative regulation of monocyte differentiation	46.95	2.45E-05	7.09E-02	4.17E-03
GO:0070227	Lymphocyte apoptotic process	6.79	3.11E-05	8.98E-02	4.99E-03
GO:0003151	Outflow tract morphogenesis	6.71	3.31E-05	9.57E-02	5.04E-03

## Discussion

Despite the high heritability estimated for AAA, GWAS studies have identified only a few significant loci (*P* < 5e-8). In an effort to further interpret the development and pathobiology of AAA disease, we integrated functional analysis of a set of SNPs in noncoding gene regulatory regions and gene coding regions by implementing cell-type-specific REs from HiChIP experiments with whole-genome sequence data. We first evaluated the HiChIP REs by measuring the consistency between their chromatin accessibility and the expression levels of their target genes. Next, we investigated these cell-type-specific REs at the network level based on the GWAS summary statistics, identifying AoSMC as the most relevant cell type for AAA. Finally, we showed that gene regulatory relations inferred from HiChIP data can be leveraged to 1) compare noncoding variants between AAA patients and healthy controls and 2) identify regulators relevant to differential gene expression between AAA patients and controls. These analyses highlighted the IL-6 pathway, *ERG*, and *KLF* as key regulators of AAA pathobiology. Although these pathways have been previously suggested to be relevant to AAA in experimental biology studies, they have not been implicated statistically in genetic studies of a large number of AAA patients and controls. Leveraging new data on the gene regulatory networks in AoSMC and HAEC, we hereby provide such statistical evidence based on the WGS data on 268 AAA individuals and various types of controls. Importantly, our analysis provides useful information on how genetic variants on cell-type-specific regulatory regions may impact the genes involved in the IL-6 pathway, *ERG*, and *KLF* regulation.

In terms of methodology, our approach differs from previous GWAS studies in its integration of reference data on cell-type-specific gene regulatory information with predicted accessibility changes due to sequence variants in personal genome data to identify disease-relevant REs, genes, and pathways. Some of the reference data in AAA-relevant cell types were generated in this study. For a complex trait other than AAA or one with limited prior knowledge, the reference high-resolution chromatin interaction data for a broad range of candidate cell types is needed to identify the most relevant cell type. With several large-scale ongoing efforts to generate genome-wide chromatin interaction data [e.g., the ENCODE project (35) and the 4D Nucleosome project (36)], we expect that the interaction data based on Hi-C, HiChIP, ChIA-PET (37), and/or promoter capture Hi-C (38) for a wider range of cell types will become available in the near future. At that time our method can be extended to handle these more complex traits.

One limitation of the current study is that there are only 268 AAA subjects with WGS data. We note the insufficient sample size and make an effort to include a larger set of control samples. We expect that as more WGS data become available for AAA our methodology will be able to generate a more comprehensive understanding of the role of regulatory variants in the genetics of AAA and highlight potentially novel mechanisms of AAA pathobiology. Moreover, it is challenging but worthwhile to comprehensively incorporate the altered TF expression caused by genetic variants into OpenCausal, which is a direction that we plan to pursue in the near future.

In summary, this study provides an avenue to decipher underlying mechanisms of disease by combining WGS data with gene regulatory relationships in relevant cell types. Our results implicate the IL-6 pathway, *ERG*, and *KLF* in the regulation of AAA biology with strong statistical significance, furthering the understanding of AAA pathogenesis as well as identifying potential therapeutic targets.

## Materials and Methods

### Cell Culture.

HAEC (catalog no. CC-2535) and human AoSMC (catalog no. CC-2571) were obtained from Lonza. The HAEC were grown in endothelial growth medium (EGM medium), which is formulated by mixing the contents of the EGM SingleQuots kit (Lonza catalog no. CC-4133 containing bovine brain extract [BBE], ascorbic acid, hydrocortisone, epidermal growth factor [hEGF], fetal bovine serum [FBS], and gentamicin/amphotericin-B [GA] with EBM basal medium (Lonza catalog no. CC-3121). AoSMC were grown in SmGM-2 BulletKit (Lonza catalog no. CC-3182). The following growth supplements are added to a 500-mL bottle of smooth muscle cell basal medium: hEGF, 0.5 mL; insulin, 0.5 mL; hFGF-B, 1 mL; FBS, 25 mL; GA-1000, 0.5 mL. Both cell types were cultured at 37 °C in 5% CO_2_. For cell culture expansion, trypsin/EDTA (ethylenediaminetetraacetic acid) was used for detachment of cells. Cells between passage 5 and 7 were used for the following HiChIP experiments.

### HiChIP.

We followed the HiChIP protocol published by Mumbach et al. (39, 40), using antibody to H3K27ac (Abcam, ab4729) with the following modifications. The cells were disassociated with 0.25% trypsin/EDTA and washed with 1× phosphate-buffered saline. Approximately 5 million HAEC or AoSMC were cross-linked with freshly prepared 1% formaldehyde and quenched with a final concentration of 125 mM glycine. For each cell line, we used ∼15 million cells (three tubes) for HiChIP. The pellet was resuspended in 500 μL of ice-cold Hi-C Lysis buffer. After digestion with 25 U (5 μL of 5U/μL) MboI restriction enzyme (NEB, R0147) and ligation, the nuclear pellet was brought up to 880 μL of nuclear lysis buffer. Samples were sheared using a Covaris E220 using the following parameters: fill level = 10, duty cycle = 5, PIP = 140, cycles/burst = 200, time = 4 min, then clarified by centrifugation for 15 min at 16,100 rcf at 4 °C. The samples were precleared with 30 μL of Dynabeads Protein A (Thermo Fisher, 10001D) at 4 °C for 1 h. We then added 3.75 μg of H3K27ac antibody to the samples and incubated overnight at 4 °C. Chromatin–antibody complexes were subsequently captured with 30 μL of Dynabeads Protein A. Approximately 2 to 9 ng of ChIP DNA was obtained following Qubit quantification. The amount of Tn5 used and number of PCR cycles performed were based on the post-ChIP Qubit amounts, as described previously in the HiChIP protocol. The library was sequenced on Illumina HiSEq. 4000 with 75-bp paired-end reads. The H3K27ac HiChIP of naïve T primary cell (CD4^+^) was downloaded from Gene Expression Omnibus (GEO) with accession no. GSE101498.

We processed the raw FASTQ files of HiChIP with HiC-Pro (41) using reference genome GRCh37/hg19. Then, hichipper (42) was employed to perform bias-corrected peak calling (i.e., anchors) and library quality control. We further applied FitHiChiP (43) to perform DNA loop calling (i.e., identification of significant contacts), where we used the bias-corrected peaks defined from hichipper as input, set the length of fixed-size genomic windows/bins as 5 kb, and selected the stringent model for inferring the background model. Finally, the loops with a *q*-value less than 0.01 were obtained for HiChIP analysis. For the noncoding variant region-based analysis, we further split the REs into 1 kb in length based on the OpenCausal setting.

### RNA-seq and ATAC-seq.

RNA-seq data of human aortic smooth muscle cells was downloaded from GEO using accession no. GSE78528, of which the gene FPKM (fragments per kilobase of exon model per million mapped reads) expression was used for AoSMC analysis (35). Human thoracic aorta endothelial cell RNA-seq data were from downloaded from GEO using accession no. GSE78613, of which the gene FPKM expression was used for HAEC analysis (35). The RNA-seq data of CD4^+^, alpha-beta T cell was from ENCODE with ID ENCSR545MEZ, of which the gene FPKM expression was used for CD4^+^ T cell analysis (44). If a gene was not represented in the RNA-seq data, it could be due to the fact that it is not expressed, or it is expressed but is not detected because of the limited sample size. For our analysis, we assumed its expression to be zero (Table 1). The ATAC-seq data of human aortic endothelial cell was downloaded from GEO using accession GSM3067767, of which the ATAC peaks were used for HAEC analysis (45). The ATAC-seq data of human coronary artery smooth muscle cells was downloaded from GEO using accession no. GSM1876025, of which the ATAC peaks were used for AoSMC analysis (46).

### Whole-Genome Sequencing Data.

High-coverage (average 50×) short-read WGS data were obtained from 268 AAA patients and 133 controls from VA Palo Alto Health Care System, Stanford University, and Kaiser Permanente as previously described (23).

### RSS-NET Network Enrichment.

Given a HiChIP-based RE-TG network, we modified our previously developed genetic effect size distribution (12, 47) as follows:

$$\beta_{j}\;∼\;\pi_{j}\;N(0,\sigma_{j}^{2})\;+\;(1-\pi_{j})\;\delta_{0},$$

$$\pi_{j}=\theta_{0}+a_{j}\theta ,$$

$$\sigma_{j}^{2}=\sigma_{0}^{2}+a_{j}\sigma^{2},$$

where $\beta_{j}$ denotes the true genetic effect of SNP j, $a_{j}=1$ if SNP j is within ±50 kb of any RE or TG in the given network and 0 otherwise. For the near-gene control network, $a_{j}=1$ if SNP j is within ±50 kb of any protein-coding gene. Following our previous work (12), we placed grid-based priors on the hyperparameters θ_0_, θ, σ_0_^2^, and σ^2^ and estimated them from data.

We combined the genetic effect size distribution above with a multiple regression likelihood based on GWAS summary statistics (47). We fitted the resulting Bayesian hierarchical model on the published European-ancestry GWAS (9) summary statistics of 1,216,709 genome-wide autosomal SNPs (minor allele frequency >1%), using an efficient variational inference algorithm (48).

For each network (either the context-specific RE-TG or the near-gene control), we summarized the enrichment strength as BF comparing the estimated marginal likelihood of an enrichment model against the estimated marginal likelihood of a baseline model ($M_{0}:\;\theta =0\;\mathrm{and}\;\sigma^{2}=0$). In this study we considered three enrichment models ($M_{1}:\;\theta >0\;or\;\sigma^{2}>0$; $M_{11}:\;\theta >0\;\mathrm{and}\;\sigma^{2}=0$; $M_{12}:\;\theta >0\;\mathrm{and}\;\sigma^{2}>0$).

The software implementing this method is freely available at https://github.com/SUwonglab/rss-net (12).

### Quantification of Gene Mutation Burden.

To predict the deleteriousness effect of each nonsynonymous SNV, we applied a similar strategy to Li et al. (23), which was based on the average performance of three algorithms [VEST3 (30, 31), MetaLR (32), and M-CAP (33)]. We averaged the prediction scores to assess the deleteriousness of each nonsynonymous SNV. Then, for each protein-coding gene we calculated the cumulative effects of nonsynonymous SNVs for each of 17,443 protein-coding genes. In detail, for gene *i* in sample *m*, we defined its mutation burden *g* as

$$g_{im}=\overset{n_{im}}{\underset{j=1}{\sum }}s_{\mathrm{ijm}},$$

where *s_ijm_* is the average deleteriousness score for SNV *j* in sample *m* and *n_im_* is the count of rare nonsynonymous SNVs on gene *i* in sample *m*. For 268 AAA patients and 133 controls, we repeated the above procedure and then obtained the deleteriousness scores for genes with a 17,443 × 401 matrix.

## Supplementary Material

Supplementary File

Supplementary File

Supplementary File

Supplementary File

Supplementary File

## Data Availability

All raw and processed HiChiP data of AoSMC and HAEC from this study have been deposited in the NCBI Gene Expression Omnibus (GEO; https://www.ncbi.nlm.nih.gov/geo/) with accession no. GSE178598.

## Change History

January 22, 2022: The byline has been updated.
